# Discussion on Microwave-Matter Interaction Mechanisms by *In Situ* Observation of “Core-Shell” Microstructure during Microwave Sintering

**DOI:** 10.3390/ma9030120

**Published:** 2016-02-23

**Authors:** Wenchao Liu, Feng Xu, Yongcun Li, Xiaofang Hu, Bo Dong, Yu Xiao

**Affiliations:** 1 CAS Key Laboratory of Mechanical Behavior and Design of Materials, Department of Modern Mechanics, University of Science and Technology of China, Hefei 230026, China; liuwc@mail.ustc.edu.cn (W.L.); huxf@ustc.edu.cn (X.H.); dongbo@mail.ustc.edu.cn (B.D.); xiaoyuxy@mail.ustc.edu.cn (Y.X.); 2 Department of Mechanics, Taiyuan University of Technology, Taiyuan 030024, China; liyongcun@tyut.edu.cn

**Keywords:** microwave sintering, microstructure formation mechanism, synchrotron radiation computed tomography, metal, ceramic

## Abstract

This research aims to deepen the understanding of the interaction mechanisms between microwave and matter in a metal-ceramic system based on *in situ* synchrotron radiation computed tomography. A special internal “core-shell” microstructure was discovered for the first time and used as an indicator for the interaction mechanisms between microwave and matter. Firstly, it was proved that the microwave magnetic field acted on metal particles by way of inducing an eddy current in the surface of the metal particles, which led to the formation of a “core-shell” microstructure in the metal particles. On this basis, it was proposed that the ceramic particles could change the microwave field and open a way for the microwave, thereby leading to selective heating in the region around the ceramic particles, which was verified by the fact that all the “core-shell” microstructure was located around ceramic particles. Furthermore, it was indicated that the ceramic particles would gather the microwaves, and might lead to local heating in the metal-ceramic contact region. The focusing of the microwave was proved by the quantitative analysis of the evolution rate of the “core-shell” microstructure in a different region. This study will help to reveal the microwave-matter interaction mechanisms during microwave sintering.

## 1. Introduction

In recent decades, microwave sintering has been developed as an environmentally friendly method for the quick preparation of high-performance metals, ceramics and composite materials [1,2,3,4,5,6]. For example, Roy *et al.* indicated that the modulus of rupture of a microwave-processed Fe-Ni sample was 60% higher than the conventional samples [1]. The superior performance of microwave-sintered products comes from the excellent microstructures, such as finer grain size and round-edged porosities [1,7,8]. These excellent microstructures are the results of the interaction between microwave and matter [7,9,10]. Therefore, once the interaction mechanisms between microwave and matter are revealed, it will hopefully be possible to regulate the performance of the product. However, microwave sintering is a complex and variable process, because not only does microwave act on matter, but matter also influences the microwave in return [11,12,13,14,15,16]. For example, Birnboim *et al.* indicated that the sintering neck would lead to the focusing of the microwave field [17]; Rybakov *et al.* showed that the direction of the microwave electric field could influence the shape of the pores [18]. The interaction of microwave and matter makes the sintering process variable, and brings great challenge for the study of microwave sintering mechanisms. Although microwave sintering is complex, it is essentially the microstructure evolution and densification process of materials driven by the microwave field [19]. At present, the microwave field cannot be obtained in micro-scale, but the evolution of the internal microstructure is the visualization of the microwave effect. Therefore, tracking the evolution of the internal microstructure and combining it with simulation or theory analysis is an effective method for researching the microwave-matter interaction mechanisms. However, owing to the extreme environment of microwave sintering (high temperature and intense radiation), it was difficult for conventional observation methods, such as SEM and TEM, to carry out *in situ* observation of internal microstructure. Different from conventional methods, the synchrotron radiation computed tomography (SR-CT) technique is a nondestructive internal detecting technique which can achieve real-time, three-dimensional (3D) and high-resolution observation under extreme environments [5,20,21]. By applying the SR-CT technique, the 3D internal microstructure at different sintering times can be directly and continuously observed without disturbing the sintering process. The validity of the SR-CT method has been verified by the comparison between SR-CT results and SEM results [22]. Based on the SR-CT experimental data, the evolution of internal microstructure can be quantitatively obtained, which makes it possible to analyze the microwave-matter mechanisms.

The main aim of this paper was to investigate the microwave-matter interaction mechanisms, meaning microwave sintering mechanisms related to the microwave response of materials, based on the *in situ* SR-CT observation of the microwave sintering process of a metal-ceramic mixed system (Ti-SiO_2_). In the experiment, we first observed an interesting phenomenon where some metal particles developed a “core-shell” structure, which was used as an indicator for the microwave-matter interaction mechanisms. The main conclusions were that: 1. it was proved that the microwave field heated metal particles by inducing an eddy current in the surface of metal particles; 2. it was proposed that the ceramic particles opened a way for the microwave and led to the redistribution of power loss; 3. it was indicated that the ceramic particles would gather the microwave and might lead to the microwave focusing on the metal-ceramic contact region.

## 2. Materials and Methods

**Figure 1:**
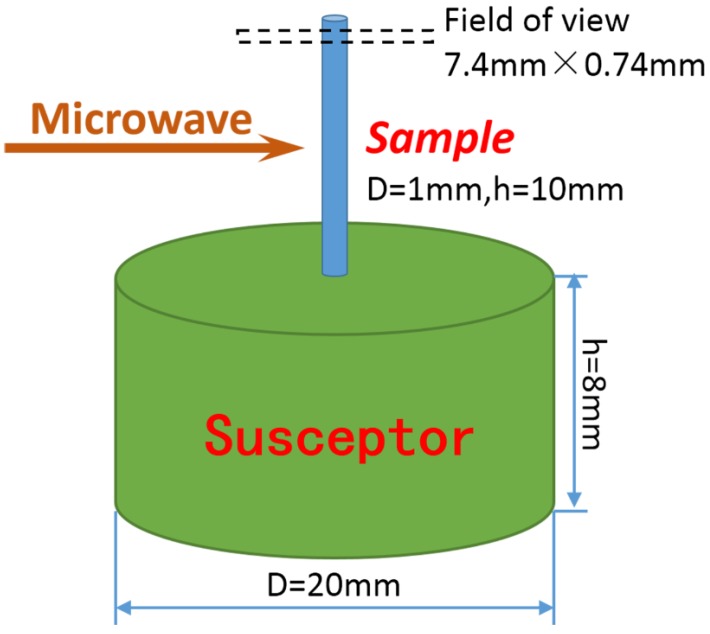
The schematic diagram of susceptor and sample.

**Figure 2:**
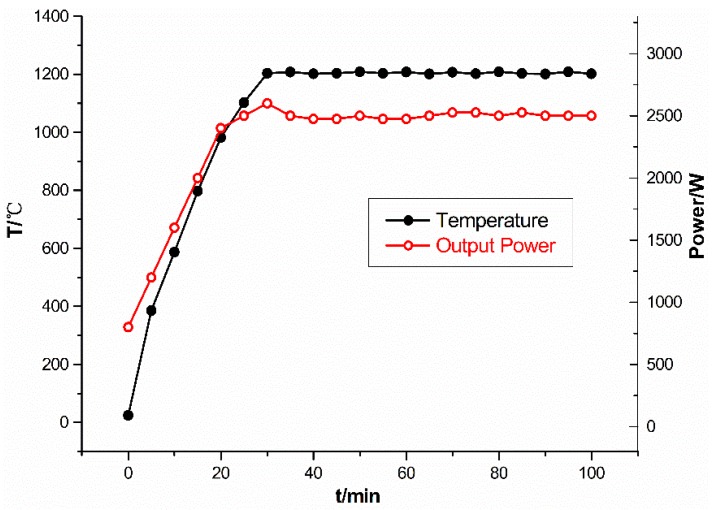
Typical temperature and output power profile.

The microwave sintering experiment of Ti-SiO_2_ was carried out on the BL13W1 beam line at Shanghai Synchrotron Radiation Facility (SSRF, Shanghai, China). The spatial resolution used in the experiment was 3.7 μm. In the experiment, titanium (purity 99.5%, average diameter: 50 μm) and silica (purity 99.9%, average diameter: 120 μm) were mixed uniformly according to the volume ratio of 3:1. Ti and SiO_2_ were chosen because they had different X-ray absorption coefficients, therefore they would have different grayscale in tomograms and could be distinguished. The mixture was uniaxially pressed into a 1 mm diameter and 10 mm height cylinder; the relative green density of the sample is 50.72%. Then the sample was introduced into a specially designed microwave furnace (multimode cavity 2.45 GHz, output power 0–3 kW). The SR-CT experiment system was shown elsewhere [23]. The cavity was replenished with helium to prevent oxidation throughout the experiment. A small cylinder silicon carbide susceptor, as shown in Figure 1, was used to assist heating [24]. The diameter of susceptor is 20 mm, and the height of susceptor is 8 mm. As the microwave power was 2500 W in the experiment, which was much larger than the absorbing power of the susceptor, the sample could be directly heated by microwave. In addition, in order to further avoid the possible influence of susceptor on the microstructure evolution process, the testing region in SR-CT experiment was selected at the top of sample which was the far end from susceptor. The temperature was controlled at 1200 °C by adjusting the output power and measured by an infrared thermal imager (type TH5104, NEC corp., Tokyo, Japan, temperature measurement range −10–1500 °C, accuracy ±1.0% (full scale), emissivity 0.50). The typical temperature and output power profile is shown in Figure 2.

## 3. Results

**Figure 3:**
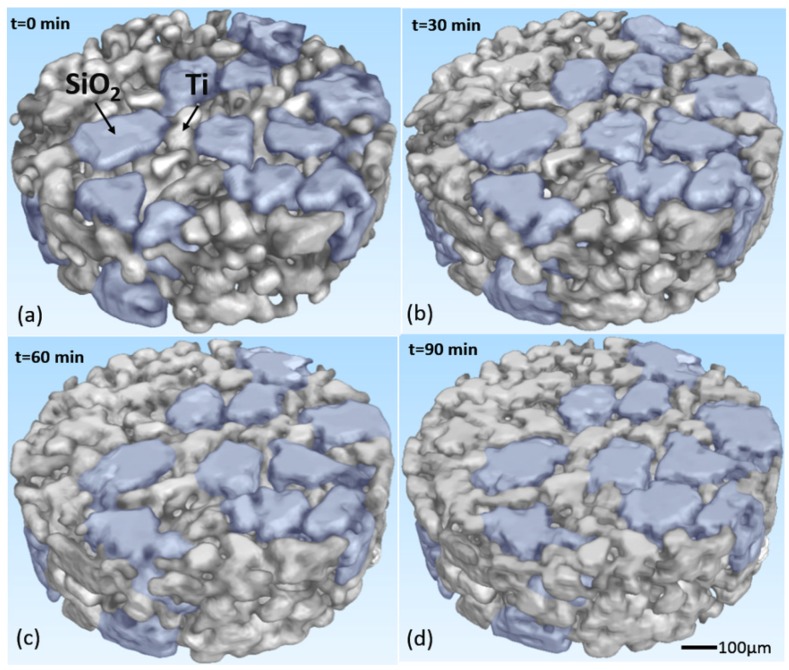
The 3D images of the sample at different times; SiO_2_ particles were marked in blue and Ti particles were gray. (**a**) t = 0 min; (**b**) t = 30 min; (**c**) t = 60 min; (**d**) t = 90 min.

Based on the SR-CT technique, 3D images of the microstructure of the same region at different times were obtained and shown in Figure 3. In this figure, the microstructure evolution was clearly observed, and usual sintering phenomena could be seen, such as the formation and growth of the sintering neck, the movement and incorporation of particles, and the densification of the sample. On the surface, everything was normal, as observed in the conventional methods [2,25].

**Figure 4:**
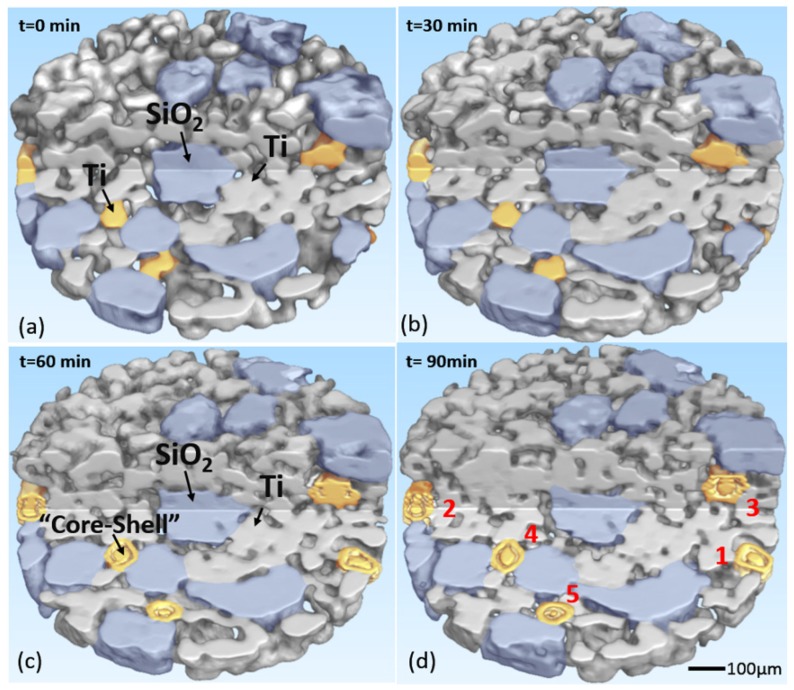
Internal microstructure of the specimen at different times, with the “core-shell” particles marked in yellow. Owing to the spatial movement of particles, particle 1 was overlapped in images (**a**) and (**b**), and the ceramic particle marked in blue over it was cut off in images (**c**) and (**d**).

**Figure 5:**
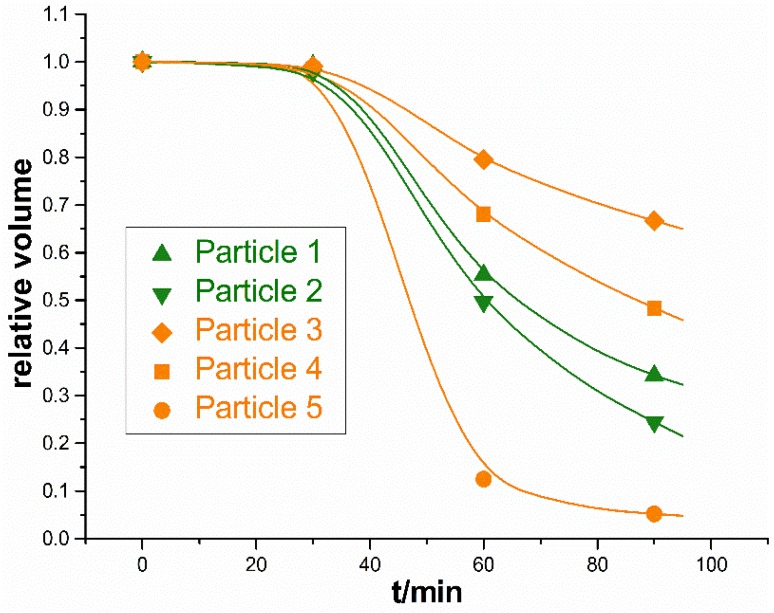
The core relative volume *versus* sintering time curves.

Fortunately, the SR-CT method can observe the internal microstructure, which is important to the study of sintering mechanisms. Figure 4 shows the internal microstructure of the specimen at different times. In this figure, a part of the sample was cut out from the 3D images, and a typical internal region was shown. It could be clearly seen that some metal particles developed a special “core-shell” microstructure, and these unique particles were marked in yellow. In the field of view, 1756 titanium particles and 36 silica particles were observed; 14 titanium particles developed a “core-shell” structure, about 0.80% of the titanium particles. The “core-shell” microstructure was located in the internal region of the particles, where it could not be observed by conventional observation methods such as optical microscopy and SEM. During the microwave sintering process, these metal particles were separated into external shell and internal core, and then the core shrank or even nearly disappeared, while the shell remained unchanged. A quantitative analysis of the evolution rate of the “core-shell” microstructure was carried out. Considering that the volume of the “core-shell” particles decreased as parts of them melted, the growth progress of the “core-shell” particles could be represented by the relative volume, the ratio of the current volume and initial volume of the particles, which were shown in Figure 5. In Figure 5, it was shown that particles in different regions had different shrink rates. Particles 1 and 2 were both in the external region of sample, and they had a similar and higher shrink rate. As for particles 3, 4, 5, they were all in the internal area of sample, but particle 5 had an ultrafast shrink rate. Why did this strange phenomenon take place?

## 4. Discussion

Microwave sintering is a complex process in which microwave and matter influence each other, and this brings about the unique advantages of optimizing microstructure and improving performance. The formation of “core-shell” microstructure must be the result of the interaction of microwave and matter. In this section, the “core-shell” microstructure was regarded as the indicator for the interaction mechanisms between microwave and matter. Through investigating the formation, distribution and evolution rate of the “core-shell” microstructure, the microwave-matter interaction mechanisms were studied. In Section 4.1, the microwave eddy current heating mechanism of metal is verified by analyzing the formation of the “core-shell” microstructure. In Section 4.2, changes in the microwave field caused by ceramic particles are discussed through analyzing the distribution and evolution rate of the “core-shell” microstructure.

### 4.1. The Effect of Microwave on Metal (Titanium) Particles

In the specimen, it was noticed that all the “core-shell” particles were metal particles. The shape of the “core-shell” microstructure was reminiscent of the microwave eddy current heating mechanism of metal. Considering the special active chemical characteristics of titanium particles and the fact that there might be an interfacial reaction between Ti and SiO_2_ [25,26], the Ti particles might owe oxide shells. If the surface of the Ti particles melted while the oxide shell remained [13,14], then the “core-shell” microstructure would appear. In order to confirm this assumption, the finite element method (FEM) was used and a model of a titanium metallic particle covered by an oxide shell was built. Considering that the diameter of the particle (50 μm) is much smaller than the wave length of the microwave (122.4 mm), the initial applied microwave field was regarded as a uniform plane electromagnetic wave in the model [19]. The frequency of the microwave is 2.45 GHz. The diameter of the particle was 50 μm, and the thickness of the oxide shell was 3 μm. The parameters used in the model are shown in Table 1. In this model, the transmission of microwaves obeys wave equations derived from the Maxwell equations [27,28]:

(1) $$\nabla^{2}\vec{H}+\omega^{2}\mu \varepsilon \vec{H}=0$$

(2) $$\nabla^{2}\vec{E}+\omega^{2}\mu \varepsilon \vec{E}=0$$

where $\vec{E}$ is the electric vector; $\vec{H}$ is the magnetic vector; *ω* is the angular frequency; *μ* is the complex permeability; *ε* is the complex permittivity. The power loss of the metal particle follows the following formula [29,30]:

(3) $$P=\frac{1}{2}\int^{\text{​}}\vec{E}\cdot {\vec{J}}_{S}dV=\frac{1}{2}\sigma \int^{\text{​}}{\left|{\vec{J}}_{S}\right|}^{2}dV$$

where *σ* is the conductivity; ${\vec{J}}_{S}$ is the surface current which is calculated by ${\vec{J}}_{S}=\vec{n}\times {\vec{H}}_{t}$; and ${\vec{H}}_{t}$ is the tangential magnetic vector. From this formula it can be seen that the power loss of the metal particle is mainly determined by the magnetic field. In the metallic particles, the alternating magnetic field induces a rotational electric field, which in turn drives the eddy current that generates heat. So the distribution of the eddy current within this particle-pore system will directly influence the heating of the metallic particle.

**Figure 6:**
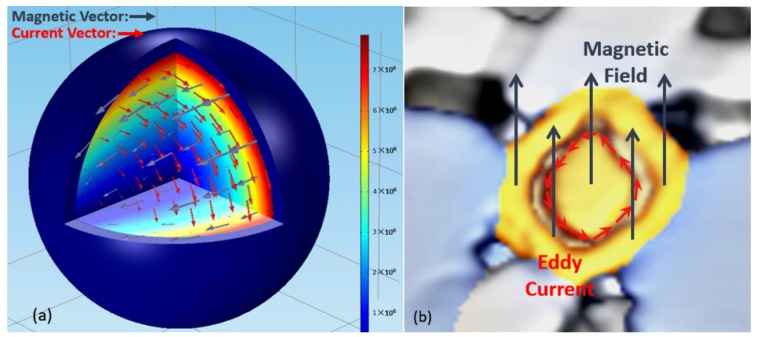
(**a**) The current in the metal particle with oxide shell; (**b**) The “core-shell” microstructure in metal particle.

From Figure 6a it is seen that the high-frequency alternating magnetic field induced the eddy current on the surface of the metal particle, and an illustration of the “core-shell” structure is shown in Figure 6b, in which the “core-shell” particle is particle 4 in Figure 4d. Furthermore, it can be seen that the eddy current on the surface was one order of magnitude higher than on the center. Therefore, the power loss on the surface was about two orders of magnitude larger, and the surface was selectively heated by the eddy current. This heterogeneous power loss would a generate large temperature gradient, and the temperature of the surface of metal particles might be much higher than the measured external temperature. Therefore, local melting occurred on the surface, and the “core-shell” microstructure formed.

The microwave eddy current heating mechanism of metal led to the formation of the “core-shell” microstructure. In other words, the “core-shell” microstructure was important evidence for the microwave eddy current heating mechanism of metal. Moreover, the formation of the “core-shell” microstructure required a local high temperature, which could only be generated by the microwave directly. Therefore, the “core-shell” microstructure was only located where the effect of the microwave was stronger, so the “core-shell” microstructure was regarded as the indicator of the microwave effect in the following discussion.

### 4.2. The Effect on Microwave by Introduction of Ceramic (Silica) Particles into Metal System

#### 4.2.1. “Microwave Passage” Effect Generated by Introduction of Ceramic (Silica) Particles

**Figure 7:**
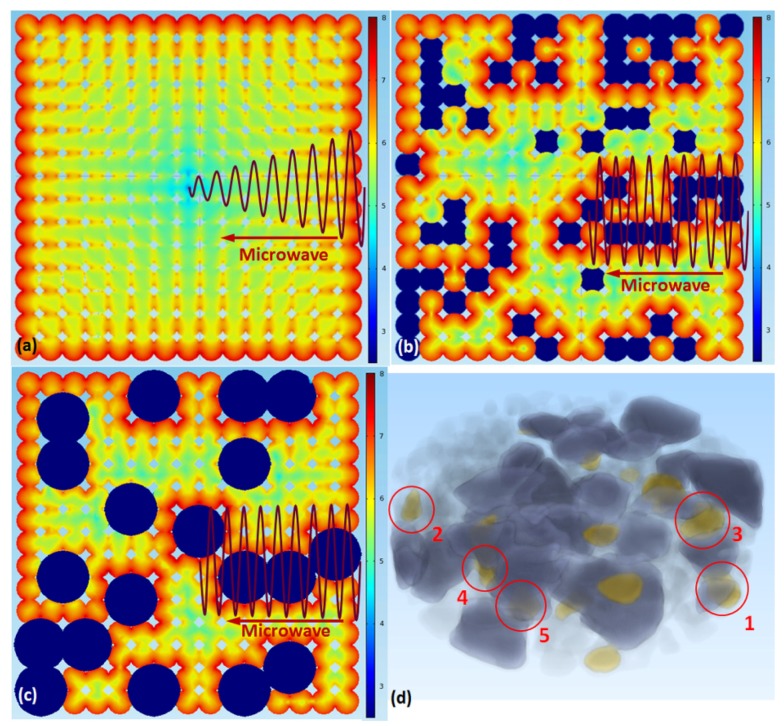
(**a**) The power loss in the pure metal sample; (**b**) The power loss in the metal-ceramic mixed sample; (**c**) The power loss in the metal-ceramic mixed sample with larger ceramic particles, which was quite similar to (**b**); (**d**) The perspective drawing of sample. The value in the color bar in (**a**), (**b**) and (**c**) was the common logarithm of the actual result considering visual effect. The yellow particles in the circle in (**d**) were the same particles shown in Figure 4.

From Figure 7d, it can be seen that the “core-shell” phenomenon only occurred in the area where the metallic particles were near the ceramic. Why do not all the metal particles grow into the “core-shell” microstructure? The introduction of ceramic particles might play an important role in the formation of the “core-shell” microstructure. As we know, the absorption, transmission and reflection of the microwave in ceramic is different from that in metal, and the microwave heating mechanisms of metal and ceramic are also distinct. Therefore, the introduction of ceramic particles might change the microwave field and influence the adjacent particles. In this regard, the model consisting of 15 × 15 particles with a sintering neck including 100% metallic particles as well as another model consisting of 15 × 15 particles including 75% metallic particles and 25% randomly distributed ceramic particles were built. In addition, a model consisting of larger ceramic particles, whose diameter was 120 um, was built as a comparison. In these models, the major aim was to study the influence of ceramic particles on the microwave field, so the oxide shells were ignored in consideration of the amount of computations. In these models, the diameter of the particles was 50 μm, the distance between two neighboring particles was 45 μm, and the other parameters were same as in the model in Section 4.1 and shown in Table 1. The results are shown in Figure 7a,b. It is important to note that silica is a typical low-dielectric-loss material under 2.45 GHz microwave radiation, so the power loss of silica particles can be ignored in general, and the ceramic particles were marked in blue in the images.

From Figure 7a it can be seen that once the connection between particles formed, the currents in the particles influenced each other. At the initial stage of sintering, there was only mechanical contact between particles. However, as the sintering process went on, the sintering neck between the particles formed, and the eddy current would be induced on the particles’ surfaces and flowed between particles. So the whole sample could be regarded as a huge particle, and only the external particles were heated by the eddy current, while the internal particles might be only heated by heat conduction. However, in the metal-ceramic sample, because of the low conductivity of ceramic, the penetration depth of the microwave in ceramic is much larger than in metal, so the microwave could spread to the middle part of the sample along the ceramic passage. Additionally, the microwave would induce a large eddy current in the metal particles adjacent to the ceramic particles, as shown in Figure 7b. Here we define it as the “microwave passage” effect. So the internal metallic particles around the ceramic could be heated by the electromagnetic wave directly. Consequently, the power loss will be quite different in these particles, and a huge temperature gradient would appear in these particles. Thus, these particles had the potential to develop a “core-shell” microstructure. The selective distribution of the “core-shell” microstructure indicated that the introduction of ceramic particles might lead to the “microwave passage” effect and, as a result, change the final microstructure of the sample.

#### 4.2.2. “Microwave Lens” Effect of Ceramic Particles

In Section 4.2.1, the influence of the addition of ceramic particles on the power loss of the metal system was studied. The results show that the addition of ceramic particles will lead to the “microwave passage effect” and promote the spread of the microwave in the middle part of the sample. If there was only the “microwave passage” effect, the external particles would be heated by the microwave directly and the “core-shell” structure would develop faster. However, the truth is not so simple. In Figure 5, it was shown that particles in different regions had different shrink rates. The different shrink rates represented the different heating rates, which meant there were extra microwave mechanisms. In the following analysis, the local microwave field that was influenced by ceramic particles was investigated.

**Figure 8:**
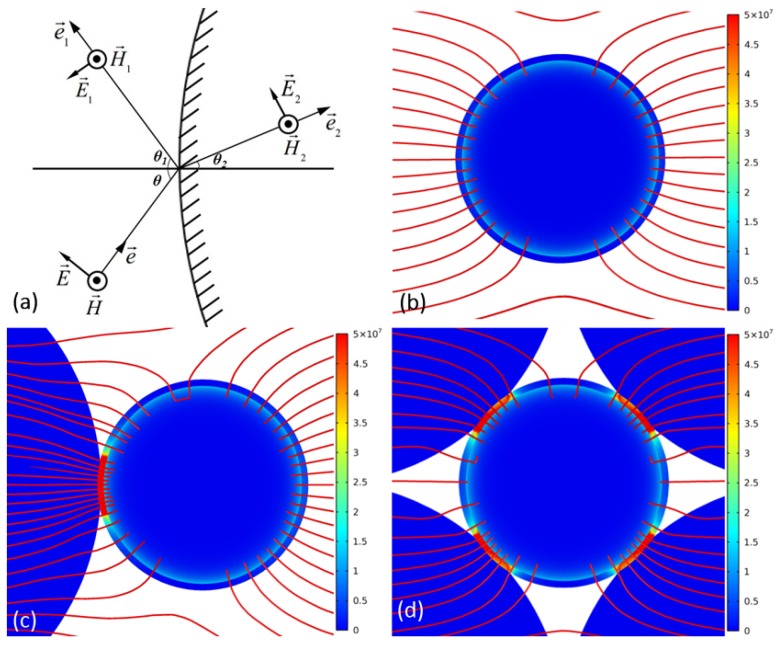
(**a**) The oblique incident electromagnetic wave in a ceramic particle; (**b**) The power loss in a metal particle covered by oxide shell, the red lines represent electric field lines; (**c**) The power loss in a metal particle that is adjacent to a ceramic particle; (**d**) The power loss in a metal particle that is surrounded by four ceramic particles.

As we know, an electromagnetic wave can travel in materials; once the electromagnetic wave passed through a heterogeneous interface, reflection and refraction happened. Assume that the applied electric vector was $\vec{E}$, as shown in Figure 8a. Then the reflected electric vector was ${\vec{E}}_{1}$ and the refracted electric vector was ${\vec{E}}_{2}$. On the outside of the ceramic particle, the total electric vector was [27,28]:

(4) $${\vec{E}}^{+}=\vec{E}+{\vec{E}}_{1}={\vec{E}}_{0}\exp\left(-j\sqrt{\mu_{1}\varepsilon_{1}}\vec{e}\cdot \vec{r}\right)+{\vec{E}}_{01}\exp\left(-j\sqrt{\mu_{1}\varepsilon_{1}}\vec{e}\cdot \vec{r}\right)$$

and on the inside of the ceramic particle, the total electric vector was:

(5) $${\vec{E}}^{-}={\vec{E}}_{2}={\vec{E}}_{02}\exp\left(-j\sqrt{\mu_{2}\varepsilon_{2}}\vec{e}\cdot \vec{r}\right)$$

where $\vec{e}$, ${\vec{e}}_{1}$, ${\vec{e}}_{2}$ represented the incident, reflected, refracted direction vectors. Then $\vec{E}$, ${\vec{E}}_{1}$, ${\vec{E}}_{2}$ were decomposed along tangential and normal. On account of that, the tangential electric vector was equal on the surface of ceramic particles, so ${\vec{E}}_{t}^{+}={\vec{E}}_{t}^{-}$ and $\theta_{1}=\theta$. Then:

(6) $$\sin\theta_{2}/\sin\theta =\sqrt{\mu_{1}\varepsilon_{1}}/\sqrt{\mu_{2}\varepsilon_{2}}$$

(7) $${\vec{E}}_{0}\cos\theta -{\vec{E}}_{01}\cos\theta ={\vec{E}}_{02}\sqrt{1-\frac{\varepsilon_{1}}{\varepsilon_{2}}\sin^{2}\theta }$$

The magnetic vectors could be analyzed in the same way. The analysis stated that refraction would happen when the microwave passed through a ceramic particle whose permittivity was larger, just like the visible light passed through glass.

Figure 8b,c showed the power loss in a metal particle that was covered by an oxide shell and in another identical metal particle that was adjacent to a ceramic particle. It could be seen that the electric lines were much more intensive in the interface between ceramic particles and the metal particle, which meant that the local electric field could greatly exceed the applied field. It indicated that the ceramic particles acted like a convex lens and would collect electric field lines within this region, which would lead to a highly non-uniform energy deposition. Here we call this phenomenon the “microwave lens” effect. As a result, the oxide shell of the metal particle would be heated fast by the huge electric field. When a metal particle was adjacent to a ceramic particle, the average power loss density of the oxide shell increased 3.9 times (from 1.14 × 10^6^ to 4.45 × 10^6^ W/m^3^). When the metal particle was surrounded by four ceramic particles, the average power loss of the shell increased to 7.00 × 10^6^ W/m^3^, about 6.1 times that of the original metal particles. The power loss of the oxide shell even exceeded the metal particle itself. As such, the “microwave lens” effect of ceramic particles made the oxide shell another heat source, so a higher temperature would be generated on the surface of metal particles. In the experiment, particles 1 and 2, located in the external area of the sample as shown in Figure 4, could be radiated by the microwave directly and they both were adjacent to one ceramic particle, so they similarly had a higher shrink rate. As for the three internal particles 3, 4 and 5, particle 3 was adjacent to one ceramic particle, and particle 4 was adjacent to two ceramic particles, so the shrink rate of particle 4 was higher than that of particle 3. Moreover, particle 5 was surrounded by several ceramic particles (some of them were cut out or covered, and can be seen in Figure 7d), thus the electromagnetic field around it was extremely large. So the shrink rate of particle 5 was even higher than those of particles 1 and 2. These results verified that the introduction of ceramic particles would influence the microwave field, and might lead to the microwave focusing on and locally heating the metal-ceramic contact region.

## 5. Conclusions

In summary, research on the interaction mechanisms between microwave and matter was carried out based on the SR-CT technique. In the experiment, the 3D images of the internal microstructure of the sample at different sintering times were constructed. An interesting “core-shell” microstructure inside metal particles was detected. The “core-shell” microstructure was used as an indicator for the microwave-matter mechanisms. Firstly, it was noticed that all the particles developing a “core-shell” structure were metal particles. The “core-shell” microstructure was likely to be the result of surface-melting. So the “core-shell” microstructure was evidence for the fact that the microwave affects metal particles by inducing an eddy current on the surface of the metal particles. Besides, it was found that the “core-shell” microstructure was located near the region of the ceramic particles. Combined with FEM simulation of current distribution in a pure metal system and metal-ceramic system, it was proposed that ceramic particles could open a way for the microwave, thereby leading to selective heating around ceramic particles. In addition, the quantitative analysis on the growth rate of the “core-shell” microstructure was carried out. It was found that the “core-shell” structure developed faster when it was surrounded by more ceramic particles. Through analyzing the local microwave absorption in a metal-ceramic contact region, it was verified that the ceramic particles could act as a lens, thereby gathering the microwave, which might lead to the microwave focusing on the metal-ceramic contact region. These results are helpful for deepening the understanding of interaction mechanisms between microwave and matter.

## Figures and Tables

**Table 1:** The parameters used in the FEM simulation.

Matter	σ (conductivity)	ε (permittivity)	μ (permeability)
SiO_2_	0	4.2 − 0.01 × i	1
Ti	2.6 × 10^6^ S/m	1	1
TiO_2_	0	10.5 − i	1
